# Morphotaxial Halogenation of Solution-Processed Two-Dimensional Indium Selenide

**DOI:** 10.1021/acs.nanolett.4c05922

**Published:** 2025-03-17

**Authors:** Brendan P. Kerwin, Jung Hun Lee, M. Iqbal Bakti Utama, Thang T. Pham, Alessandro Pereyra, Vinod K. Sangwan, Vinayak P. Dravid, Antonio Facchetti, Mark C. Hersam, Tobin J. Marks

**Affiliations:** † Department of Chemistry and the Materials Research Center, Northwestern University, 2145 Sheridan Road, Evanston, Illinois 60208-3113, United States; ‡ Department of Materials Science and Engineering and the Materials Research Center, Northwestern University, 2220 Campus Drive, Evanston, Illinois 60208-3108, United States; § International Institute for Nanotechnology, Northwestern University, Evanston, Illinois 60208, United States; ∥ School of Materials Science and Engineering, Georgia Institute of Technology, Atlanta, Georgia 30332, United States; ⊥ Department of Electrical and Computer Engineering, Northwestern University, 2145 Sheridan Road, Evanston, Illinois 60208-3113, United States

**Keywords:** 2D materials, halogenation, chemical conversion, morphotaxy, solution processing

## Abstract

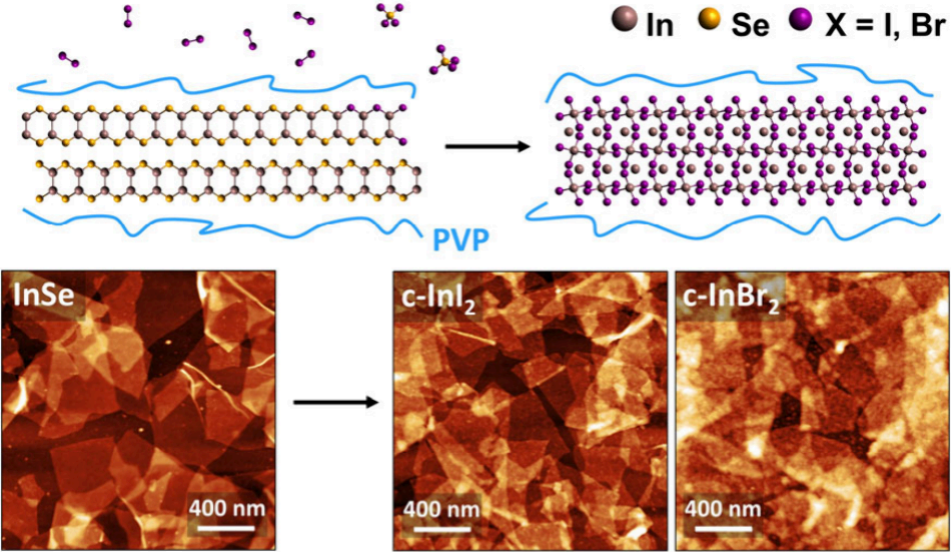

Morphotaxy, a process by which a 2D material is chemically modified while retaining its original physical dimensions, is an emerging strategy for synthesizing unconventional materials at the atomically thin limit. Morphotaxy is typically implemented by vapor-phase reactions on mechanically exfoliated or vapor-deposited 2D van der Waals (vdW) materials. Here we report a method for converting solution-processed films of 2D InSe into InI_2_ and InBr_2_ using dilute I_2_ and Br_2_ solutions, respectively. The converted materials retain the physical dimensions of the original 2D flakes, providing access to non-vdW indium halides in ultrathin form. Liquid-phase exfoliation directly enables this morphotaxial reaction by producing nanosheets with high surface areas and introducing residual polyvinylpyrrolidone that stabilizes the flake morphology and slows the reactivity of I_2_ and Br_2_. Overall, this work presents a versatile strategy for achieving atomically thin metal halides and offers mechanistic insights relevant to the morphotaxial halogenation of other solution-processed 2D materials.

Over the past two decades, the family of two-dimensional (2D) materials has expanded steadily, enabling access to diverse optical, electronic, and magnetic phenomena.^1−3^ The vast majority of 2D materials are atomically thin analogues of bulk, layered van der Waals (vdW) crystals, which are obtained either through top-down exfoliation or bottom-up growth processes. Chemical modification, including substitutional doping^4^ and surface functionalization,^5,6^ is a powerful strategy for tailoring the physical properties of 2D materials for a wide range of applications. In the extreme case, chemical modification can completely convert a 2D material into a new 2D material of different chemical composition. This class of reactions has recently been described by the term morphotaxy.^7^ The key attraction of morphotaxy is that the new material retains the shape and dimensions of the original flake or film.^7^ Morphotaxy allows materials to be accessed in an atomically thin form even if they cannot be exfoliated from a bulk crystal or grown by traditional methods–which can be the case if no bulk analogue exists,^8,9^ the bulk form does not have a layered crystal structure,^10−12^ or individual layers in the vdW crystal are difficult to isolate.^13^ Importantly, morphotaxy not only expands the scope of accessible 2D materials but also facilitates the fabrication of lateral and vertical 2D heterostructures.^14−16^

Metal chalcogenides have been widely explored as starting materials for morphotaxial transformations. These materials have well-studied methods of preparation and are amenable to a range of chemical reactions including oxidation,^14,17^ cation exchange,^18^ chalcogen exchange,^15,18^ and pnictogen substitution.^11,12^ However, an underexplored area of morphotaxy research is the halogenation of metal chalcogenides. A large number of emerging 2D materials contain metal–halogen bonds, such as the monolayer ferromagnet CrI_3_,^19−22^ the noncolinear antiferromagnet NiI_2_,^23−25^ and the antiferromagnetic anisotropic semiconductor CrSBr,^26−28^ thus motivating the morphotaxial synthesis of additional 2D metal halides. Morphotaxial halogenation of metal chalcogenides has only been reported in two cases–namely, the synthesis of BiTeX (X = Br, Cl) from Bi_2_Te_3_ using BiX_3_ vapor^13^ and Se-doped InF_3_ through a high-pressure and high-temperature reaction of InSe and XeF_2_.^10^ Therefore, it is of high interest to expand the toolbox of morphotaxial reactions to include a generalized halogenation strategy with a broadened scope of final products and properties.

The majority of reported morphotaxial reactions have been demonstrated using mechanically exfoliated or vapor-deposited starting materials. A key knowledge gap exists in the application of morphotaxy to solution-processed 2D materials, a burgeoning field that enables scalable, low-cost production of printed and flexible electronics and optoelectronics.^29−32^ Solution-processed films present more chemically complex systems than mechanically exfoliated or vapor-deposited materials since they often contain residual solvents, surfactants, and polymers as well as a large degree of morphological disorder. Due to these challenges, there have been limited demonstrations of morphotaxy using solution-processed 2D materials.^10,18^ In addition, key mechanistic questions for morphotaxial processes in solution-processed 2D materials have yet to be answered, including the role of organic residues in morphotaxial reactions and whether nanosheets in percolating films can maintain their morphologies following chemical conversion.

Here we report the morphotaxial bromination and iodination of solution-processed InSe films using dilute solutions of I_2_ and Br_2_. X-ray photoelectron spectroscopy (XPS), time-of-flight secondary ion mass spectrometry (ToF-SIMS), and energy dispersive X-ray spectroscopy (EDS) confirm that selenium is completely removed in the final material and iodine or bromine are present in an approximate 2:1 atomic ratio with indium. Raman spectroscopy provides further spectroscopic evidence for the identification of these materials as indium dihalides. In addition, atomic force microscopy (AFM) reveals that the shape and dimensions of the original InSe nanosheets are maintained through the reaction. Notably, this morphotaxial synthetic pathway allows the non-vdW materials InI_2_ and InBr_2_ to be produced in ultrathin form, although they ultimately become amorphous, as confirmed by transmission electron microscopy (TEM) and selected area electron diffraction (SAED). We also show that residual surface-bound polyvinylpyrrolidone (PVP) from the original solution-based InSe exfoliation plays a key role in mediating the reaction and stabilizing the nanosheet morphology. Furthermore, we demonstrate that In_2_Se_3_ can also be used as a starting material for this morphotaxial halogenation reaction. This work provides key mechanistic insights and establishes a morphotaxial halogenation pathway that can likely be generalized to other solution-processed 2D materials.

InSe dispersions were prepared by electrochemical exfoliation using established procedures.^33−35^ To avoid ambient oxidation of InSe, all procedures were carried out in an inert glovebox atmosphere using anhydrous solvents.^36−38^ The solution-exfoliated InSe nanosheets were characterized before treatment using AFM, TEM, and XPS. Topographical AFM images show that the exfoliated flakes have an average thickness of ∼2 nm and an average lateral size of ∼700 nm (Figure S1). Transmission electron micrographs show outlines of distinct overlapping flakes (Figure S2a), while SAED of the film shows a 6-fold diffraction pattern consistent with the hexagonal lattice symmetry of InSe (Figure S2b).^39^ XPS analysis shows In 3*d* and Se 3*d* peaks with binding energies matching those reported in literature (Figure 1b).^35^ Quantification of these peaks gives a Se:In ratio of ∼1.2. Notably, the Se 3*d* region shows no features in the range of 58–62 eV, indicating that no InSe oxidation occurs during liquid-phase exfoliation and thin film preparation. Significant carbon, nitrogen, and oxygen XPS peaks are observed due to the presence of residual PVP (Figure S3).

**Figure 1:**
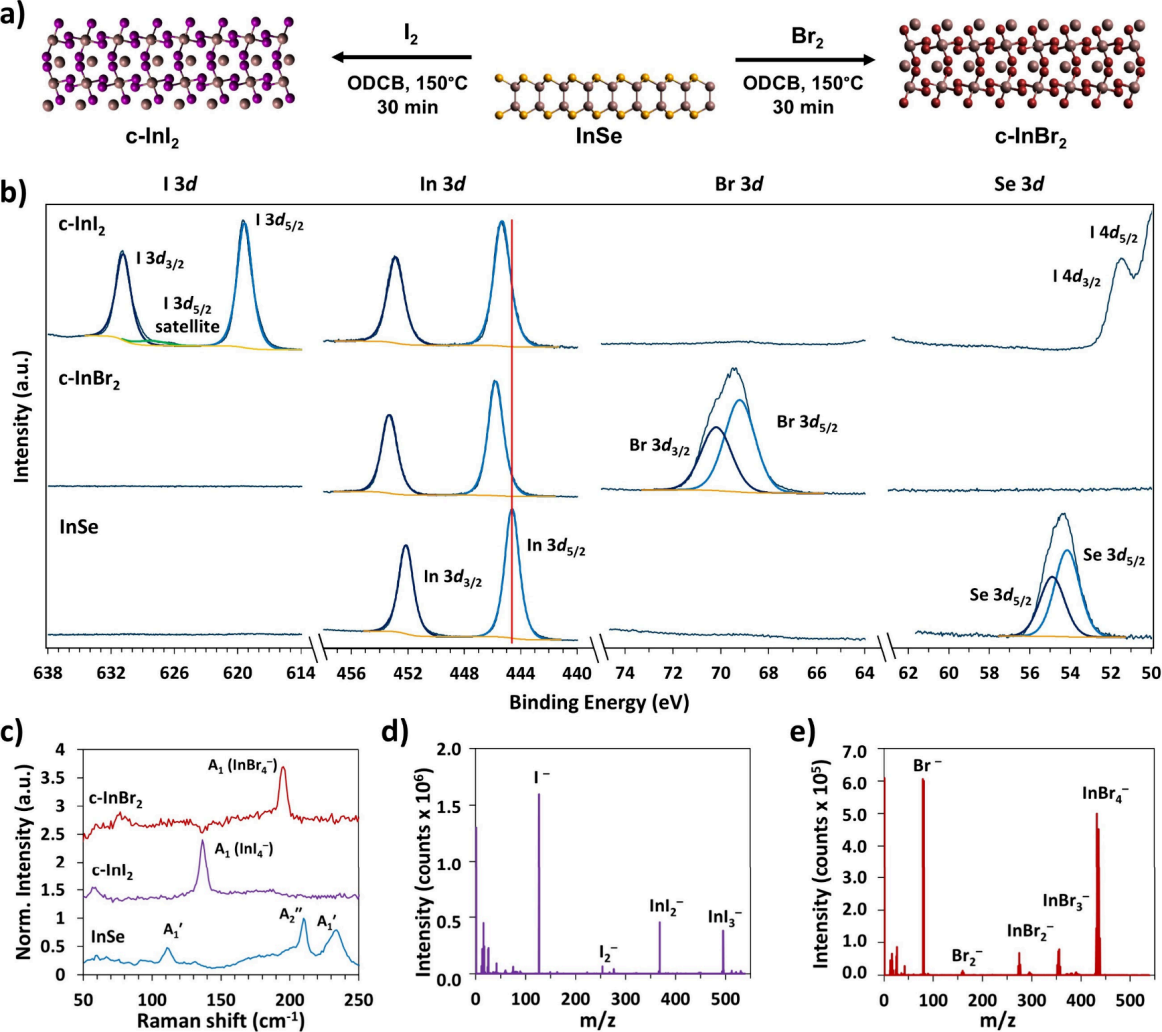
(a) Schematic representation of the morphotaxial halogenation of InSe. (ODCB = *o*-dichlorobenzene.) (b) X-ray photoelectron spectroscopy (XPS) of selected elemental peaks for electrochemically exfoliated InSe and morphotaxially converted indium halides. Binding energies are referenced to the SiO_2_ substrate (Si 2*p* = 103.5 eV). The spectra are shifted vertically for clarity, and a vertical line is included to assist in comparing the In 3*d* peak positions. (c) Raman spectra of InSe, c-InI_2_, and c-InBr_2_ films. The spectra are shifted vertically for clarity. (d, e) Time-of-flight secondary ion mass spectrometry (ToF-SIMS) of (d) c-InI_2_ and (e) c-InBr_2_ collected under negative ion detection mode.

To prepare the halogenated materials, spin-coated films of InSe were treated with dilute solutions of I_2_ and Br_2_ at 150 °C under inert atmosphere, as shown in Figure 1a. Following treatment, XPS shows the complete removal of selenium from the films and the appearance of iodine and bromine peaks (Figure 1b). The In 3*d* peak shifts from 444.6 to 445.3 and 445.8 eV for the I_2_-treated film and Br_2_-treated film, respectively. Depth profiling shows that the halogen and indium signals are present throughout the entire ∼100 nm thick film, while no selenium peaks are observed (Figure S5), indicating that our approach is effective in fully converting percolating films of InSe.

Indium forms a variety of halides with iodine and bromine, most notably trihalides, dihalides, and monohalides. The trihalides are commercially available as microcrystalline powders. InI_3_ adopts a dimeric structure consisting of In_2_I_6_ molecules,^40^ while InBr_3_ adopts a 2D layered crystal structure.^41^ The dihalides are isostructural, adopting a mixed-valent In(InX_4_) structure.^42,43^ The trihalides and dihalides are difficult to distinguish on the basis of XPS peak positions alone: both InI_3_ and InI_2_ show a single In 3*d* doublet with the 3*d*_5/2_ component centered at 445.1 ± 0.1 eV, while InBr_3_ and InBr_2_ appear at 445.8 ± 0.1 eV and 445.9 ± 0.1 eV, respectively.^44^ Therefore, our experimentally observed indium binding energy is consistent with both stoichiometries. The Br 3*d* and I 3*d* peak positions also align well with the literature values for both compounds.^44^

To assess the stoichiometry of the converted films, atomic percentages were calculated based on the XPS peak areas. The ratio of halogen to indium in the films shows a strong depth dependence. Measurements taken at the surface of the films treated with I_2_ and Br_2_ show an I:In ratio of 4.4 and a Br:In ratio of 3.6, respectively. However, the ratios fall to 2.2 and 2.3, respectively, after ion beam etching for 30 s (Figure S5). The second set of data more accurately reflects the composition of the converted material, since the surface of the film likely contains excess physisorbed I_2_ and Br_2_ that is not removed following the halogenation reaction. The chemical shifts of these species overlap strongly with those of the c-InI_2_ and c-InBr_2_, so the individual contributions to the I 3*d* and Br 3*d* signals are not resolved in the XPS data. Furthermore, the X:In ratio of approximately 2:1 is corroborated by energy dispersive X-ray spectroscopy (EDS) measurements, as discussed below. These results indicate that the reactions convert InSe to indium dihalides. We therefore denote the chemically converted materials as c-InI_2_ and c-InBr_2_.

The chemically converted c-InI_2_ and c-InBr_2_ materials, as well as the untreated InSe, were analyzed by Raman spectroscopy. The Raman peak positions of the starting material match those reported for few-layer InSe, with major peaks at 111 cm^–1^, 210 cm^–1^, and 233 cm^–1^ (Figure 1c).^45^ After halogenation, c-InI_2_ shows a single strong peak at 138 cm^–1^, while c-InBr_2_ shows a single peak at 196 cm^–1^ (Figure 1c). These features can be assigned to the symmetric stretching modes of InX_4_^–^ anions, which are present in solid InI_2_ and InBr_2_.^46−49^ For comparison, we also performed Raman spectroscopy on InBr_3_ and InI_3_ prepared by mechanical exfoliation of commercially available powders (Figures S6–S8). Mechanically exfoliated InI_3_ shows several peaks below 200 cm^–1^, the strongest of which (133 cm^–1^) corresponds to a symmetrical stretching mode of In_2_I_6_ dimers.^46^ InBr_3_ shows a major peak at 169 cm^–1^ as well as smaller peaks at 83 cm^–1^ and 56 cm^–1^ in agreement with literature.^47,48^ These results further support the assignment of the converted material as InX_2_ rather than InX_3_. However, the Raman signals for c-InI_2_ and c-InBr_2_ are relatively weak compared to the background, and other Raman peaks reported for the dihalides are not observed. These observations suggest that our materials contain the same local structures observed in InI_2_ and InBr_2_ (i.e., InX_4_^–^ ions) but lack the long-range order of those crystal structures. The low crystallinity of the converted films is confirmed by selected area electron diffraction (*vide infra*). Annealing of the c-InI_2_ and c-InBr_2_ samples does not enhance the Raman signal, but instead shows the disappearance of the InX_4_^–^ peaks above 300 °C (Figure S9). This transition is accompanied by a thinning of the films and a loss of flake morphology (Figure S10), suggesting that the halides are stable up to approximately 200 °C.

Figures 1d and 1e show the mass spectra of c-InI_2_ and c-InBr_2_, respectively, obtained using time-of-flight secondary ion mass spectrometry (ToF-SIMS). The spectra show strong halide ion (X^–^) signals as well as indium halide fragments (InX_n_^–^), which are identified by their *m*/*z* and isotope peak ratios. The presence of InX_n_ species indicates covalent bonding between indium and halogen atoms in the converted material. Notably, Se^–^ is not observed in either spectrum, corroborating the XPS results. Other indium-containing species such as InO_n_^–^ or InSe_n_^–^ are also absent.

AFM images of the c-InI_2_ and c-InBr_2_ samples show that the flake morphology is retained after conversion to the indium halides. Figures 2a–c show AFM images of drop-casted nanosheets of InSe before and after the reaction with I_2_ and Br_2_. The c-InI_2_ and c-InBr_2_ films possess clearly defined flakes of comparable size to the original InSe nanosheets. The morphology of the converted nanosheets indicates that the original InSe flakes template the growth of the halides, demonstrating a clear example of morphotaxial conversion.^7^ Since neither InI_2_ nor InBr_2_ has a layered crystal structure, it is not possible to produce ultrathin flakes of these materials by mechanical or liquid-phase exfoliation of the bulk crystals. These InX_2_ nanosheets are therefore unprecedented non-vdW 2D materials that can only be realized through morphotaxial conversion. Notably, the AFM measurements were performed in air without loss of flake morphology, indicating that the flakes are not highly hygroscopic.

**Figure 2:**
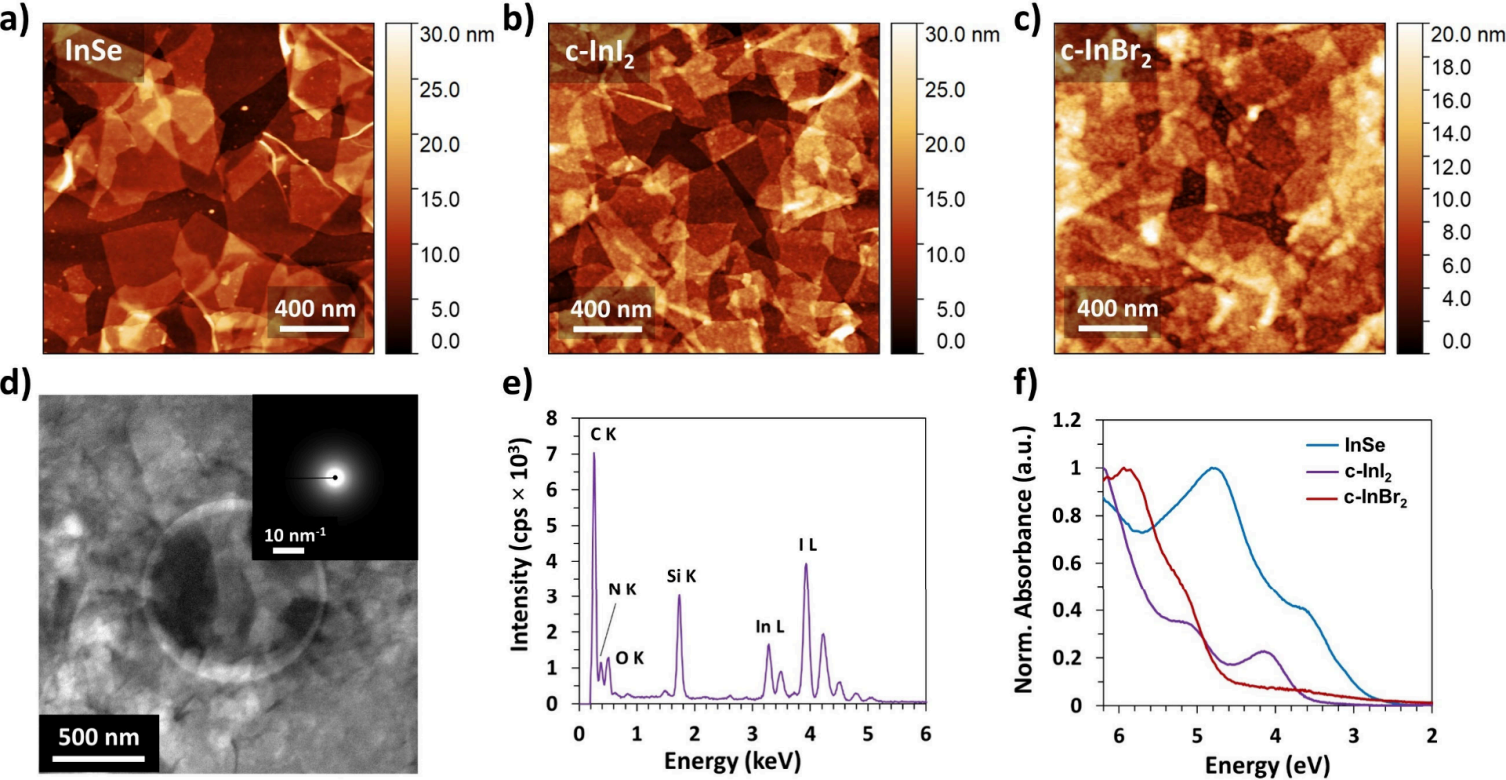
(a) Atomic force microscopy (AFM) height image of electrochemically exfoliated InSe nanosheets drop-casted on Si/SiO_2_. (b, c) AFM images of (b) c-InI_2_ nanosheets and (c) c-InBr_2_ nanosheets produced through morphotaxial conversion of InSe. Individual flakes with distinct edges are visible in all three cases. (d) Transmission electron microscopy (TEM) image of c-InI_2_ nanosheets prepared on a holey SiN_*x*_ membrane. Inset: Selected area electron diffraction (SAED) pattern showing diffuse rings. (e) Energy dispersive X-ray spectroscopy (EDS) spectrum of c-InI_2_. (f) UV–vis optical absorbance spectra of InSe, c-InI_2_, and c-InBr_2_ films prepared on CaF_2_ and encapsulated with 30 nm Al_2_O_3_ grown by atomic layer deposition.

To further assess the crystallinity and composition of the converted materials, we analyzed a c-InI_2_ film using TEM and EDS. Despite the relatively thick film, Figure 2d confirms that the film is composed of individual flakes, corroborating the AFM results. Following halogenation, the film shows diffuse rings in SAED, indicating that c-InI_2_ is amorphous (Figure 2d, inset). The amorphous nature of the flakes likely results from the ultrathin dimensions of the material. In particular, since InI_2_ is not a layered crystal, surface reconstructions are likely to disrupt the crystalline order of ultrathin flakes. Similar amorphization of non-van der Waals crystals at the 2D limit has been observed in several previous morphotaxy studies,^10,11,17^ and the mechanism of these reconstructions should be a topic for future studies. EDS measurements of c-InI_2_ indicate an I:In ratio of ∼2.3, further confirming the dihalide stoichiometry (Figure 2e). Quantification of the EDS spectrum of c-InBr_2_ similarly gives a Br:In ratio of ∼1.9 (Figure S11).

UV–vis optical absorbance measurements reveal a distinct shift in the optical properties of the films following halogenation. InSe shows absorption maxima at 3.8 and 4.8 eV (Figure 2f), whereas the halogenated materials are significantly blue-shifted. Specifically, c-InI_2_ absorbs strongly above 6 eV and has peaks at 4.1 and 5.3 eV, while c-InBr_2_ has a strong peak at 5.9 eV with a shoulder at 5.3 eV. InI_2_ and InBr_2_ are predicted to be indirect-gap semiconductors with bandgaps of 1.79 and 2.41 eV, respectively.^50^ Therefore, it is likely that the observed absorption bands correspond to higher-energy transitions, which are more favorable than absorption at the band edges due to the indirect band gap. The dimensions of the nanosheets may also be one of the contributors to the blue shift compared to bulk InI_2_ and InBr_2_, as many materials show band gap widening due to quantum confinement effects at atomically thin dimensions.

We propose the reaction scheme shown in Figure 3a, in which I_2_ or Br_2_ oxidizes selenium atoms and substitutes into the crystal lattice, forming selenium halide side products. No selenium-containing species were detected by ToF-SIMS in either the indium halide films or in drop-casted films of the reaction solutions, indicating that these side products likely decompose in solution and/or volatilize during the reaction. We hypothesize that residual PVP plays a key role in moderating the reaction. The presence of residual polymer is evident from the carbon, nitrogen, and oxygen peaks in the XPS spectra (Figure S3). PVP is known to reversibly complex with I_2_ and Br_2_, as shown in Figure 3a.^51^ PVP-I_2_ is widely used as a topical antiseptic,^51,52^ while PVP-Br_2_ has been used as a brominating agent in organic synthesis.^53,54^ In these applications, the PVP-X_2_ complex slowly releases I_2_ or Br_2_ to produce a more controlled reaction and/or longer-lasting treatment. We thus hypothesize that surface-bound PVP mediates the interaction of I_2_ and Br_2_ with the surfaces of the InSe nanosheets. To test this hypothesis, we prepared InSe dispersions and films containing no PVP and exposed them to the same reaction conditions used to produce c-InI_2_ and c-InBr_2_. AFM images show that the morphology of the original InSe flakes is completely lost during the reaction (Figure 3b–d). These results indicate that morphotaxial conversion requires a slow and controlled process enabled by the presence of PVP.

**Figure 3:**
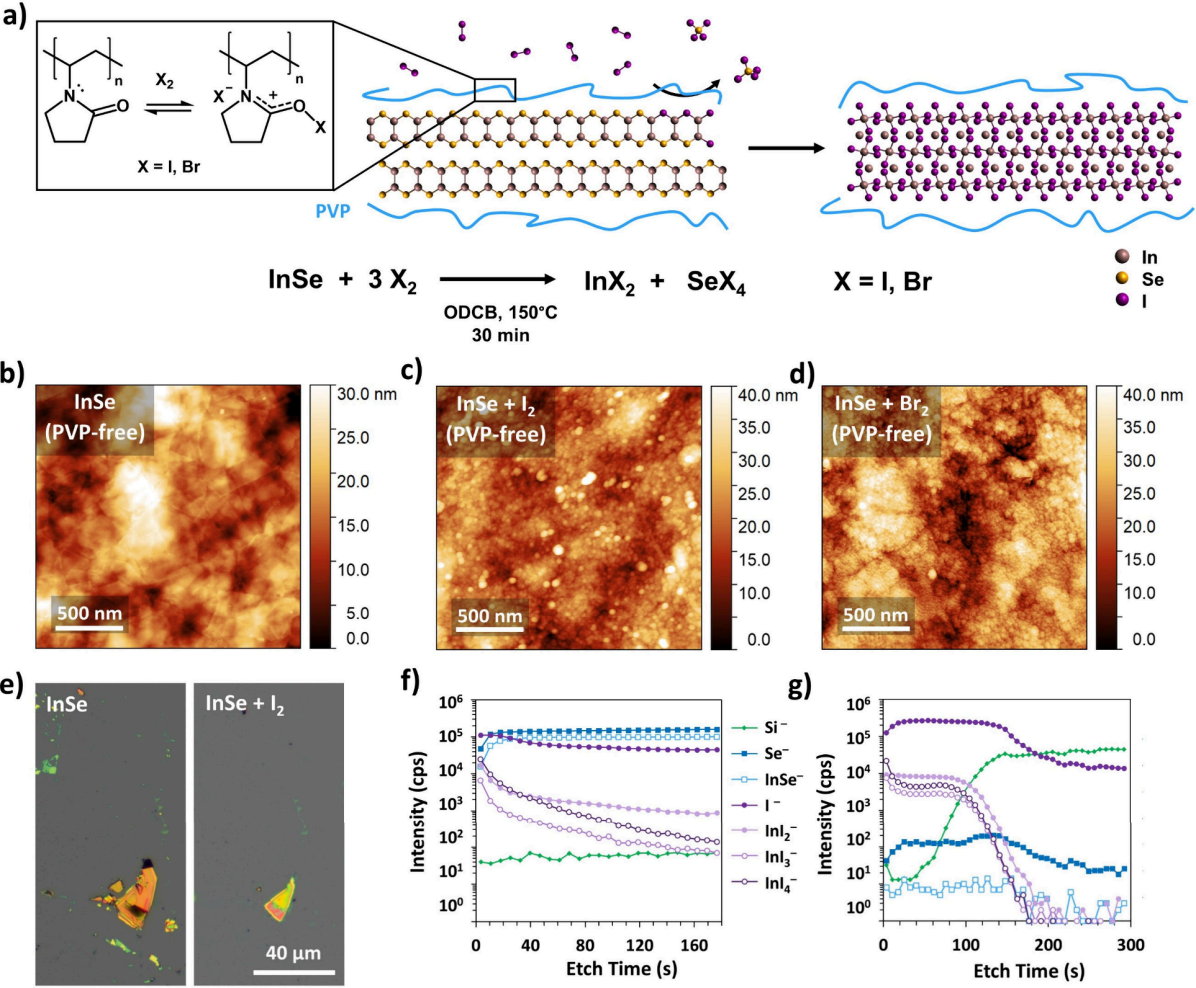
(a) Proposed reaction scheme for the morphotaxial halogenation of InSe. (b–d) AFM micrographs of PVP-free InSe films (b) as prepared and treated with (c) I_2_ and (d) Br_2_. (e) Optical micrographs of mechanically exfoliated InSe flakes before and after treatment with I_2_. Relatively thin flakes (blue/green) are removed, while thicker flakes (orange/brown) shrink in size and change color. (f) ToF-SIMS depth profile of mechanically exfoliated InSe treated with I_2_. (g) ToF-SIMS depth profile of c-InI_2_ obtained by iodination of electrochemically exfoliated InSe (with PVP).

To further explore the reaction mechanism, we attempted morphotaxial conversion of mechanically exfoliated InSe flakes. Figures 3e and S12 show InSe flakes prepared by standard Scotch tape methods under inert atmosphere. The thickness of these flakes can be roughly identified by color due to thin-film interference effects.^55^ Exposure to I_2_ or Br_2_ results in the disappearance of thinner flakes and a change in the color and lateral dimensions of thicker flakes, indicating InSe etching. In light of this observation, it is surprising that the flakes in the solution-processed films (∼2 nm thick) are not completely etched, since they are significantly thinner than the mechanically exfoliated flakes (>10 nm thick). These results suggest a dual role for PVP. First, the pyrrolidone groups bind to I_2_ and Br_2_ and slow the reaction at the flake surface, enabling controlled substitution of halogens for selenium atoms. Second, the surface-bound polymer provides mechanical support to stabilize the flake structure and prevent the newly formed indium halide from dissolving in solution.

Notably, the present halogenation reactions do not allow full conversion of the mechanically exfoliated flakes. ToF-SIMS depth profiling shows the presence of halides and indium halides on the surface of mechanically exfoliated flakes, but the intensity of these peaks falls significantly below the surface of the material (Figures 3f and S12). Meanwhile, the selenium and indium selenide peaks remain consistently high at all depths, indicating that the majority of the material remains InSe. In comparison, ToF-SIMS depth profiles of c-InI_2_ and c-InBr_2_ show significantly lower selenium and indium selenide signals but strong halide and indium halide signals throughout the thickness of the films (Figures 3g and S12). The incomplete conversion of the mechanically exfoliated flakes can be explained by the thickness of the flakes. I_2_ and Br_2_ react with the surface of the multilayered flakes and are unable to penetrate into the bulk of the material. In percolating films, however, the reagents can infiltrate through interflake gaps and completely convert the ultrathin nanosheets due to their high surface area. This observation points to a distinct synthetic advantage for solution-processed 2D materials in morphotaxial conversions–namely, a percolating film of ultrathin flakes enables complete chemical conversion even for reactions that cannot penetrate bulk crystals.

To expand the scope of the morphotaxial halogenation process, In_2_Se_3_ dispersions were prepared by electrochemical exfoliation and treated with I_2_ and Br_2_ using the same conditions as InSe. The as-prepared films show characteristic Raman peaks at 96 cm^–1^, 168 cm^–1^, and 208 cm^–1^, consistent with reported values for In_2_Se_3_.^34,56^ The Raman spectra of the halogenated films match those of c-InI_2_ and c-InBr_2_, with major peaks at 138 cm^–1^ and 196 cm^–1^, respectively (Figure 4a). As in the case of InSe, the morphology of the individual flakes in the film is retained following conversion (Figures 4b–d). XPS analysis confirms that selenium is replaced with iodine or bromine throughout the full thickness of the films (Figures S13 and S14). These results indicate that solution-phase reactions with I_2_ and Br_2_ are applicable to multiple 2D indium selenides and suggest that other 2D metal chalcogenides can be halogenated with this approach.

**Figure 4:**
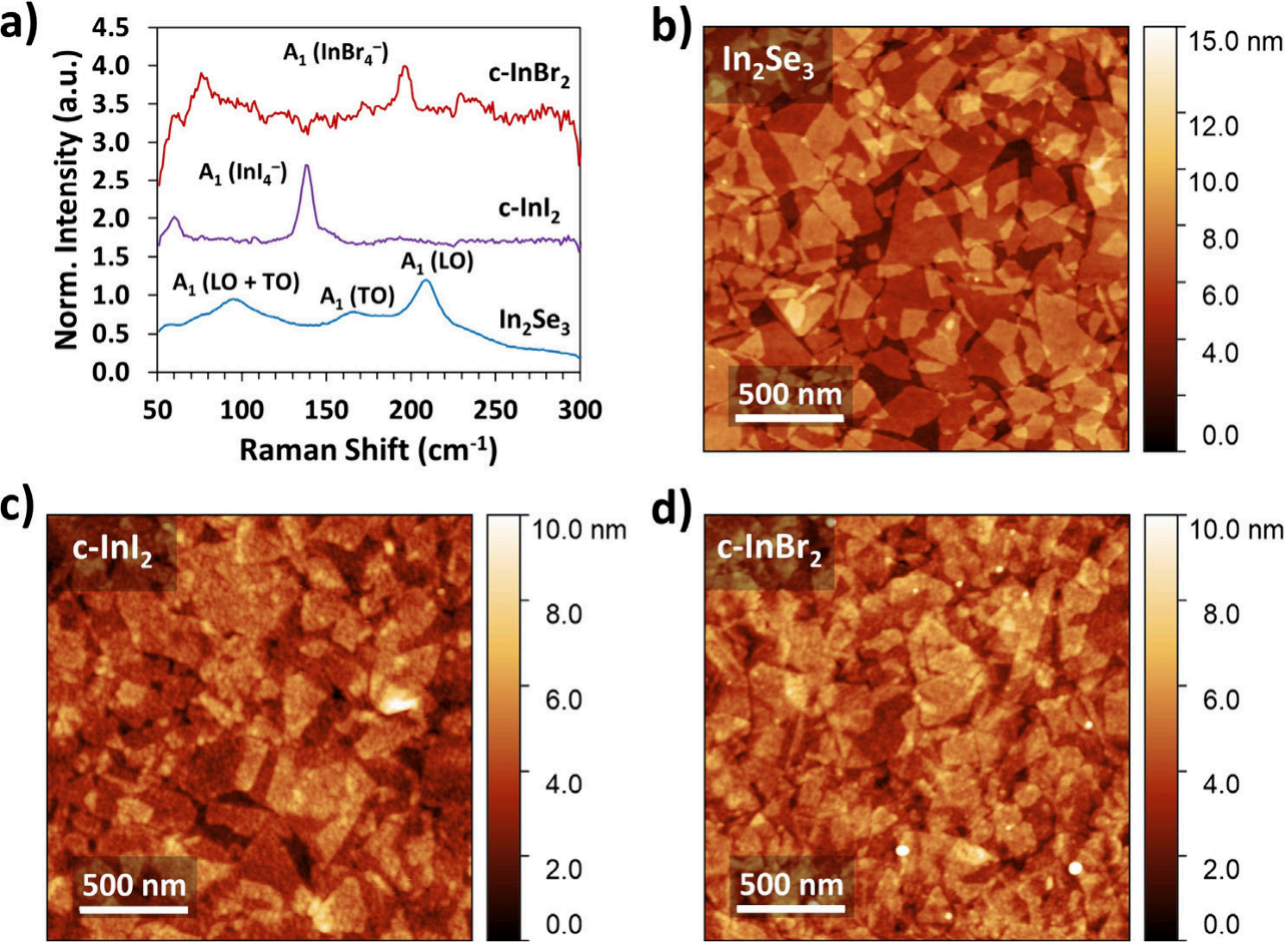
(a) Raman spectra of solution processed In_2_Se_3_ before and after reaction with I_2_ and Br_2_ (532 nm laser excitation). (b) AFM images of solution-processed In_2_Se_3_ deposited on Si/SiO_2_. (c, d) AFM micrographs of (c) c-InI_2_ and (d) InBr_2_ derived from the morphotaxial conversion of In_2_Se_3_ with I_2_ and Br_2_.

In this study, we have shown that liquid-phase exfoliated films of 2D InSe and In_2_Se_3_ nanosheets undergo morphotaxial conversion to ultrathin nanosheets of InI_2_ and InBr_2_ through a solution-based reaction with I_2_ or Br_2_. The reaction achieves complete replacement of Se^2–^ with I^–^ or Br^–^ while maintaining a flake-like morphology. These reactions thus achieve direct bromination and iodination of 2D metal chalcogenides and suggest a versatile pathway to achieving 2D metal halides. Notably, this work identifies key mechanistic considerations for the application of morphotaxy to solution-processed 2D materials. In particular, the residual polymer PVP plays a key role in mediating the substitution reaction and stabilizing the flake morphology. Furthermore, the porous structure of solution-processed percolating films consisting of ultrathin nanosheets enables complete chemical conversion, providing a distinct advantage over mechanically exfoliated flakes where the reaction is confined to the surface.

Compared to other demonstrations of morphotaxy, our pathway is notably more scalable by using solution-based processes to both produce the starting material and achieve the chemical conversion. Dozens of 2D materials have been produced through solution-based processing, providing a variety of starting substrates to test the scope of this reaction in future studies. We anticipate that each system will require slightly different reaction conditions depending on the lattice energy of the starting 2D material and the target halide. For example, a recent study reported no chemical conversion or chalcogen removal after applying the same bromination conditions to WSe_2_, WS_2_, MoSe_2_, and MoTe_2_.^57^ While reaction concentration and temperature can be easily adjusted, the identity of the stabilizing polymer provides another variable for controlling the reaction. A variety of polymers can be used to stabilize 2D materials, some of which–including poly(vinyl alcohol) (PVA) and cellulose–are also know to form complexes with iodine.^58^ The exploration of new 2D material and polymer systems are promising avenues for future research.

Device applications of indium dihalides remain largely unexplored in the existing literature. The versatile synthesis strategy for ultrathin metal halides via morphotaxy presented in this work provides a pathway to advance detailed application studies of these materials. Specifically, we propose that indium dihalides have the potential to serve as scalable solution-processed dielectric materials, which can provide high performance even without crystalline structures. Furthermore, our morphotaxial halogenation approach could facilitate future studies on magnetic 2D metal halides such as CrI_3_ and NiI_2_ which are difficult to produce through traditional solution processing methods.

## Supporting Information Available

Additional experimental details and characterization of solution-processed InSe, c-InI_2_ and c-InBr_2_ films and mechanically exfoliated InI_3_ and InBr_3_ (PDF)

## Abbreviations

THAB: tetraheptylammonium bromide
PVP: polyvinylpyrrolidone
ACN: acetonitrile
DMF: dimethylformamide
IPA: isopropyl alcohol
ODCB: *ortho*-dichlorobenzene. AFM, atomic force microscopy
XPS: X-ray photoelectron spectroscopy
ToF-SIMS: time-of-flight secondary ion mass spectrometry
TEM: transmission electron microscopy
SAED: selected area electron diffraction
EDS: energy dispersive X-ray spectroscopy
