# Supplementary material for: Morphotaxial Halogenation of Solution-Processed Two-Dimensional Indium Selenide
Source: Nano Lett. 2025 Mar 17;25(12):4734–42. doi: 10.1021/acs.nanolett.4c05922 (PMC11951151; doi:10.1021/acs.nanolett.4c05922)
Supplement: Supplementary file 1 — nl4c05922_si_001.pdf [file nl4c05922_si_001.pdf]

Supporting Information

Morphotaxial Halogenation of Solution-Processed Two-Dimensional Indium Selenide

Brendan P. Kerwin,[a‡] *Jung Hun Lee,*[b‡] *M. Iqbal Bakti Utama,*[b,c‡] *Thang T. Pham,*[b] *Alessandro Pereyra,*[b] *Vinod K. Sangwan,*[b] *Vinayak P. Dravid,*[b,c] *Antonio Facchetti,*[a,c,d] *Mark C. Hersam,*[a,b,c,e] *Tobin J. Marks*[a,b,c,*]

[a] Department of Chemistry and the Materials Research Center, Northwestern University, 2145 Sheridan Road, Evanston, IL 60208-3113, United States.

[b] Department of Materials Science and Engineering and the Materials Research Center, Northwestern University, 2220 Campus Drive, Evanston, IL 60208-3108, United States.

[c] International Institute for Nanotechnology, Northwestern University, Evanston, IL 60208, United States.

[d] School of Materials Science and Engineering, Georgia Institute of Technology, Atlanta, GA 30332, United States.

[e] Department of Electrical and Computer Engineering, Northwestern University, 2145 Sheridan Road, Evanston, IL 60208-3113, United States.

* Corresponding author: Tobin J. Marks (t-marks@northwestern.edu)

TABLE OF CONTENTS

1. **Experimental Methods S3**

2. **Characterization of Solution-Processed InSe..S6**

3. **XPS Depth Profiling of c-InI_2 and c-$InBr_2$ S8**

4. **Analysis of Commercial InI_3 and $InBr_3$...S9**

5. **Thermal Stability of c-InI_2 and c-$InBr_2$..S10**

6. **TEM and EDS Characterization of c-$InBr_2$ S12**

7. **Halogenation of Mechanically Exfoliated InSe S12**

8. **Morphotaxial Halogenation of In_2Se_3 ....S13**

1. Experimental Methods

General Considerations

All reagents were purchased from commercial sources and used without further purification unless otherwise noted. All materials were prepared and processed under inert glove box atmosphere (H_2O < 0.1 ppm, O_2 < 0.1 ppm) unless otherwise noted.

XPS data were measured using a Thermo Scientific NEXSA G2 system equipped with an Al K-Alpha X-ray source. A flood gun was applied to mitigate charging effects during measurements. XPS data were analyzed using Avantage software using standard fitting procedures. Each region of interest was fit with a smart background. Peaks were fit using a 75% Gaussian/Lorentzian line shape where the peak width and tail component were left as optimization parameters. Doublet peaks were constrained with matching peak widths and known height ratios provided in Avantage. AFM images were recorded in ambient conditions with an Asylum Cypher AFM in standard tapping mode using a NanoWorld NCHR-W Si cantilever (resonant frequency ≈ 320 kHz). AFM data were analyzed using Gwyddion software, where plane leveling and row alignment were applied to each image. ToF-SIMS data were acquired using an IONTOF M6 system and analyzed using SurfaceLab 7 software. Raman spectra were measured using Horiba XPlora Plus and Horiba LabRAM HR Evolution systems equipped with a 532 nm laser and analyzed with LabSpec 6 software. To minimize the effect of ambient and laser-induced oxidation, Raman samples were sealed into an air-free stage (Linkam, HFS350EV-PB4) inside a nitrogen glove box. In the case of commercial InI_3 and $InBr_3$, mechanically exfoliated flakes dissolved even when sealed in the Linkam stage. For more stringent air exclusion, the InI_3 and $InBr_3$ Raman samples were loaded inside a chip carrier (Spectrum Semiconductor Materials, CSB02824) that was capped with a glass coverslip and sealed using epoxy (Ossila, E132) inside a nitrogen glove

box. Optical absorption spectroscopy was conducted using an Agilent Cary 5000 UV-Vis-NIR spectrophotometer. Transmission electron microscopy (TEM) data were collected on an aberration-corrected JEOL ARM200 S/TEM operating at 200 kV. Energy dispersive X-ray spectroscopy (EDS) data were acquired at 200 kV with Dual SSD EDS detector (1.7 sr). Data processing (background subtraction, signal mapping and elemental quantification) was conducted using Gatan GMS software.

Preparation of InSe and In_2Se_3 Samples

Bulk crystals of InSe and In_2Se_3 (American Elements, 99.999% purity) were electrochemically intercalated by applying a bias at -5 V (10 min) and then -10 V (110 min) while immersed in a 5 mg/mL solution of tetraheptylammonium bromide (THAB) in acetonitrile (ACN). Next, the intercalated crystals were exfoliated by bath sonication (40 kHz) in a 22.5 mg/mL solution of polyvinylpyrrolidone (PVP, average MW ≈ 29 kDa) in N,N-dimethylformamide (DMF, 99.8% anhydrous) for 90 min. The exfoliated dispersions were then centrifuged at 32 krpm (125,755 × g) for 90 min, after which the PVP/DMF supernatant was removed and replaced with isopropyl alcohol (IPA, 99.5% anhydrous). The centrifugation step was repeated to further remove residual PVP/DMF. After these washing steps, 5 mL of IPA was added to the collected pellet before bath sonicating for 10 min, after which the dispersion was centrifuged at 7.5 krpm (5386 × g) for 5 min to remove unexfoliated material. By combining dispersions from multiple crystals during the solvent exchange step, a high-concentration final dispersion (1-2.5 mg/mL) was obtained. InSe and In_2Se_3 films (~100 nm thickness) were prepared by spin coating (30 sec at 4 krpm) on Si/SiO_2 substrates (300 nm thermal oxide) or other substrates as noted. To achieve thinner or sub-monolayer films for AFM and TEM/EDS measurements, the InSe or In_2Se_3

dispersion was diluted and drop-casted on the chosen substrate. All procedures were carried out in an inert glovebox atmosphere (H_2O < 0.1 ppm, O_2 < 0.1 ppm) using anhydrous solvents.

Halogenation of InSe and In_2Se_3 Films

Stock solutions of I_2 and Br_2 were prepared in anhydrous *o*-dichlorobenzene (ODCB) and degassed by three freeze-pump-thaw cycles before use. Spin-coated thin films (~100 nm thick) of InSe or In_2Se_3 flakes were immersed in dilute solutions of I_2 or Br_2, and then heated at 150 °C for 30 min. Typical procedures used 250 μM I_2 and 1 mM Br_2 solutions since a higher concentration of Br_2 was needed to achieve full conversion compared to I_2. After the sample was removed from the halogenation solution, excess liquid was removed by briefly spinning the sample on a spin-coater. All procedures were carried out under inert atmosphere.

Exfoliation of Commercial InI_3 and $InBr_3$

Microcrystalline powders of InI_3 (Thermo Fisher, 99.999%), and $InBr_3$ (Sigma-Aldrich, 99.999%) were used to prepare mechanically-exfoliated flakes. It was found that these materials can be exfoliated by standard Scotch-tape methods in a nitrogen-filled glovebox. A small amount of the powder was placed onto the adhesive side of Scotch tape. The best results were obtained by selecting larger flakes of the powder with visible crystalline facets. The flakes were crushed with a spatula and then repeatedly peeled apart using Scotch tape before being transferred onto Si/SiO_2. In the case of InI_3, the final samples only have flake-like morphology if exfoliated and transferred rapidly, likely due to a reaction with the tape adhesive (Figure S6). For both InI_3 and $InBr_3$, the flakes immediately form droplets when exposed to air or when kept in poorly sealed containers (Figure S7).

2. Characterization of Solution Processed InSe

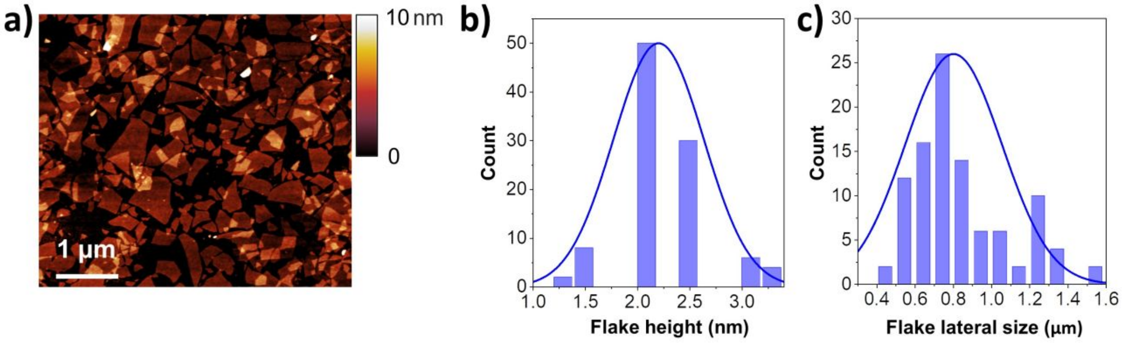

Figure S1. AFM image and flake dimensions for electrochemically exfoliated InSe. (a) Height image of the flakes drop-casted onto Si/SiO_2. (b) Histogram of flake heights. (c) Histogram of lateral flake widths.

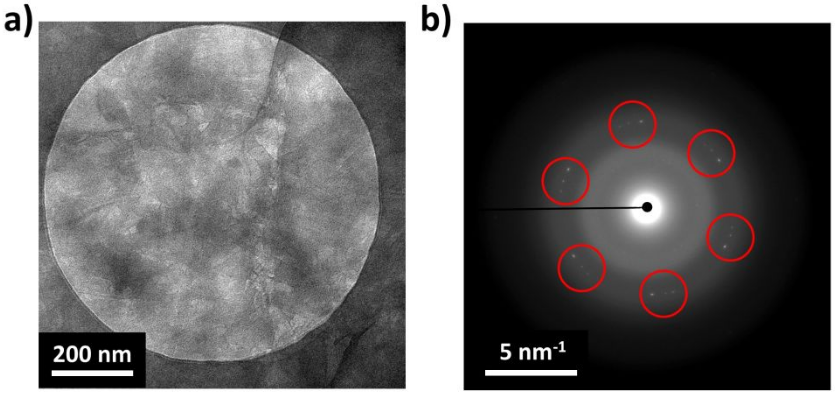

Figure S2. (a) TEM micrograph of InSe nanosheets deposited on a holey SiN_x membrane. (b) SAED pattern of the InSe film.

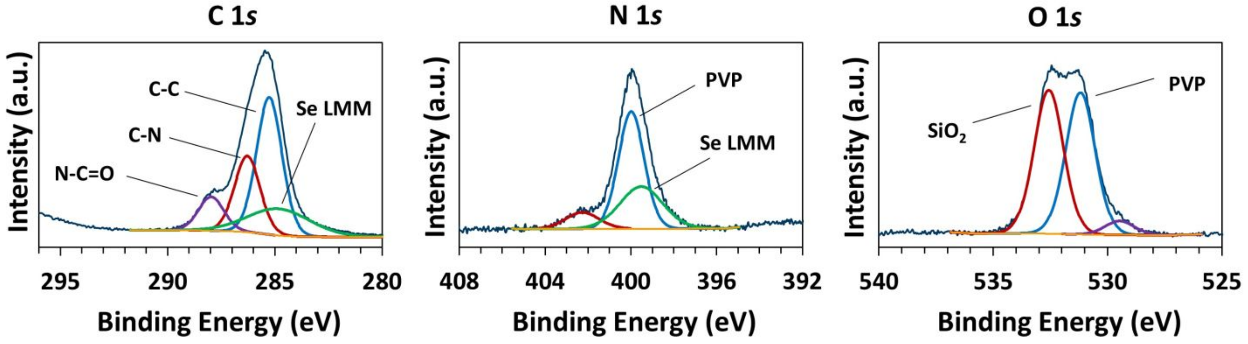

Figure S3. Carbon, nitrogen, and oxygen XPS spectra of electrochemically exfoliated InSe. All binding energies are referenced to the Si 2*p* signal from the SiO_2 substrate (Si 2*p* = 103.5 eV).

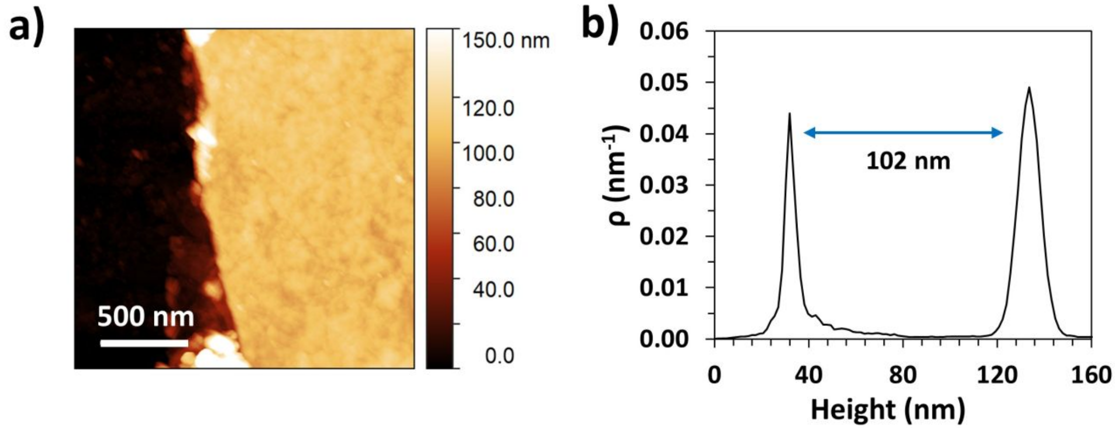

Figure S4. (a) AFM height image of a spin-coated InSe film measured along the edge of a scratch in the film to determine film thickness. (b) Height distribution for the sample in (a). The two peaks represent the height of the substrate and the film, giving a film thickness of 102 nm.

3. XPS Depth Profiling of c-InI_2 and c-$InBr_2$

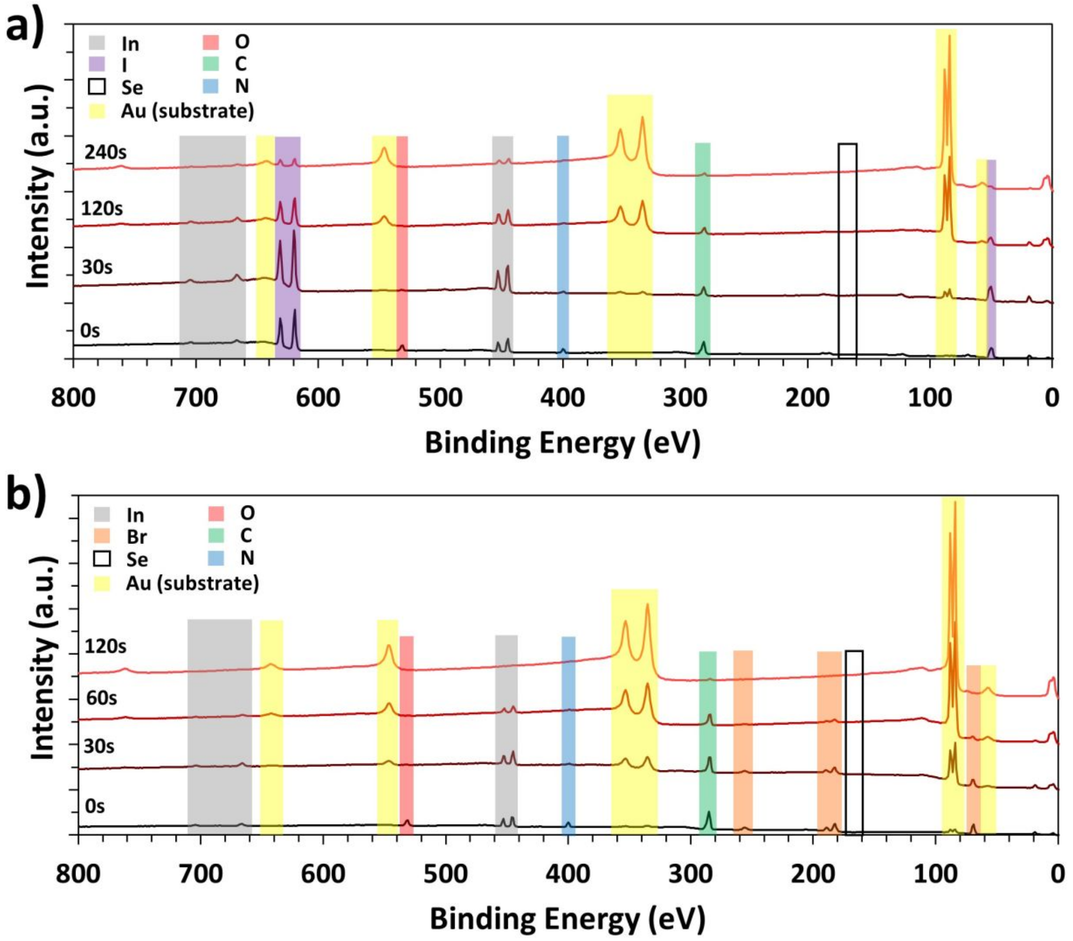

Figure S5. XPS depth profiles of (a) c-InI_2 and (b) c-$InBr_2$. Binding energy scales are referenced to the substrate, which is an evaporated Au film on Si/SiO_2 (Au $4f_{7/2}$ = 84.0 eV). The spectra are shifted vertically for clarity.

4. Analysis of Commercial InI_3 and $InBr_3$

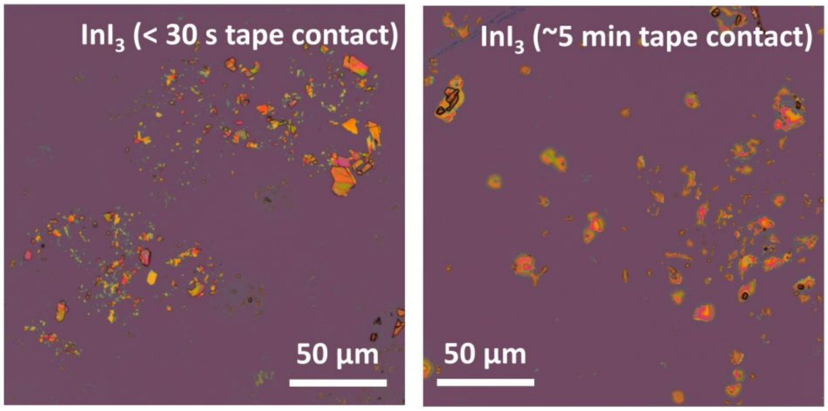

Figure S6. Optical micrographs of InI_3 samples obtained through Scotch tape exfoliation of commercial powders. Flakes obtained by exfoliating and transferring quickly (<30 s) show flake-like morphology with faceted sides. When left in contact with tape adhesive for too long (~5 min), InI_3 flakes show a "melted" appearance around their edges due to reaction with the adhesive.

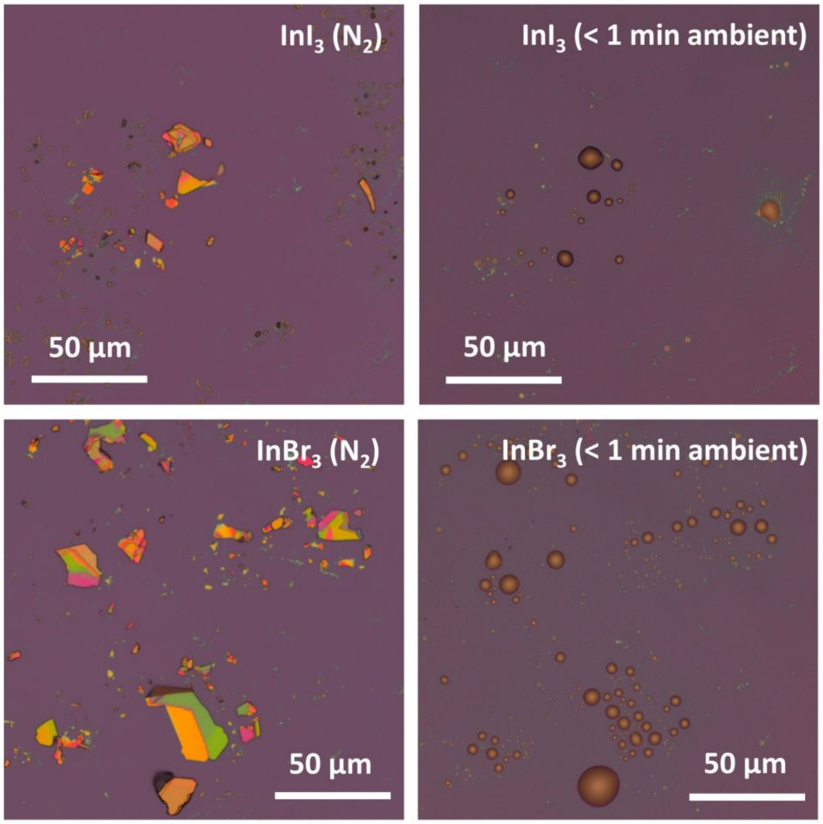

Figure S7. Optical micrographs of InI_3 and $InBr_3$ flakes obtained through mechanical exfoliation. Exfoliation under nitrogen atmosphere gave clearly defined flake shapes (left), while exposure to ambient atmosphere resulted in the formation of droplets (right).

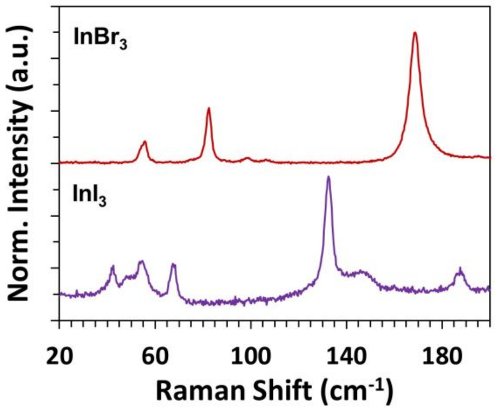

Figure S8. Raman spectra of exfoliated InI_3 and $InBr_3$ flakes measured under inert atmosphere. Spectra are normalized to the main sample peaks at 132 cm^{-1} and 170 cm^{-1}, respectively. The spectra are shifted vertically for clarity.

5. Thermal Stability of c-InI_2 and c-$InBr_2$

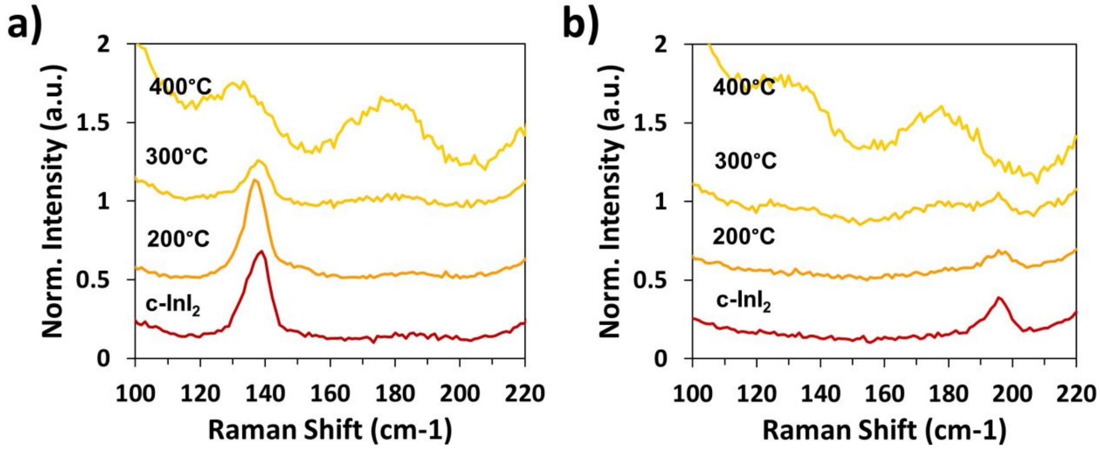

Figure S9. Raman spectra of (a) c-InI_2 and (b) c-$InBr_2$ following annealing up to 400 °C. The spectra are normalized to the silicon substrate peak at 301 cm^{-1} and shifted vertically for clarity.

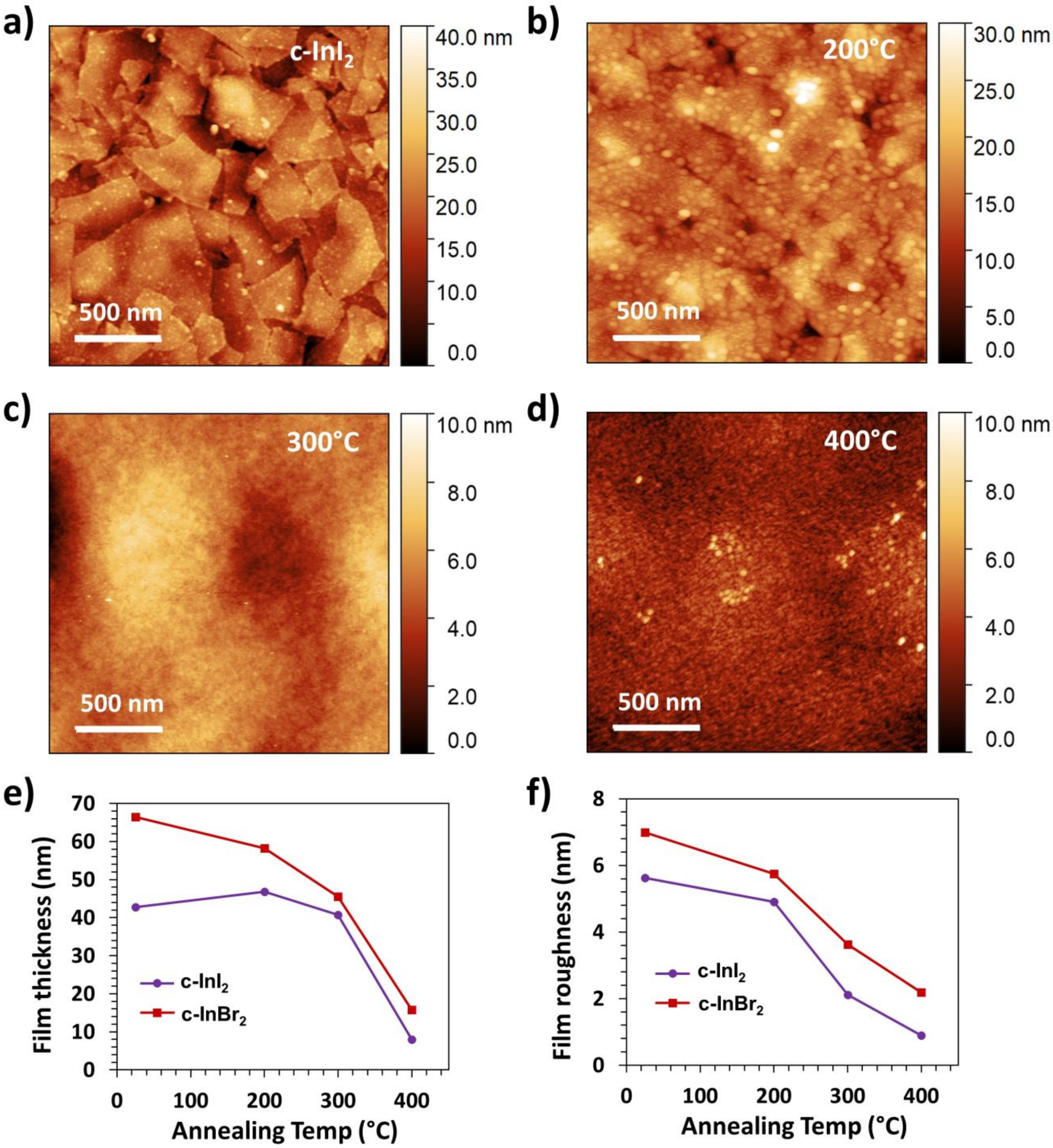

Figure S10. (a-d) AFM images of c-InI_2 as prepared and annealed to 200 °C, 300 °C, or 400 °C for 1 hour under inert conditions. (e) Film thickness of c-InI_2 and c-$InBr_2$ following annealing. Film heights were obtained by scratching the film and measuring AFM along the edge of the scratch. (f) Film roughness measurements of c-InI_2 and c-$InBr_2$ following annealing.

6. TEM and EDS characterization of c-$InBr_2$

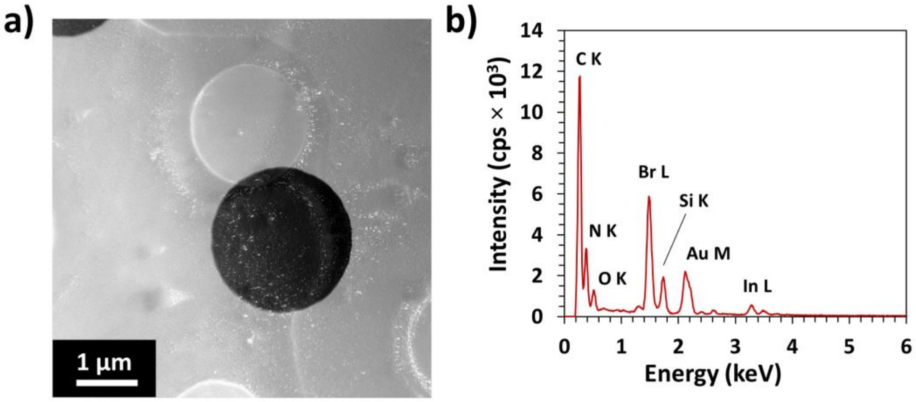

Figure S11. (a) TEM micrograph of c-$InBr_2$ flakes on a holey SiN_x membrane.(b) EDS of the c-$InBr_2$ film. Gold nanoparticles appear on the surface of the material due to sample contamination, resulting in the Au peak in the EDS spectrum.

7. Halogenation of Mechanically Exfoliated InSe

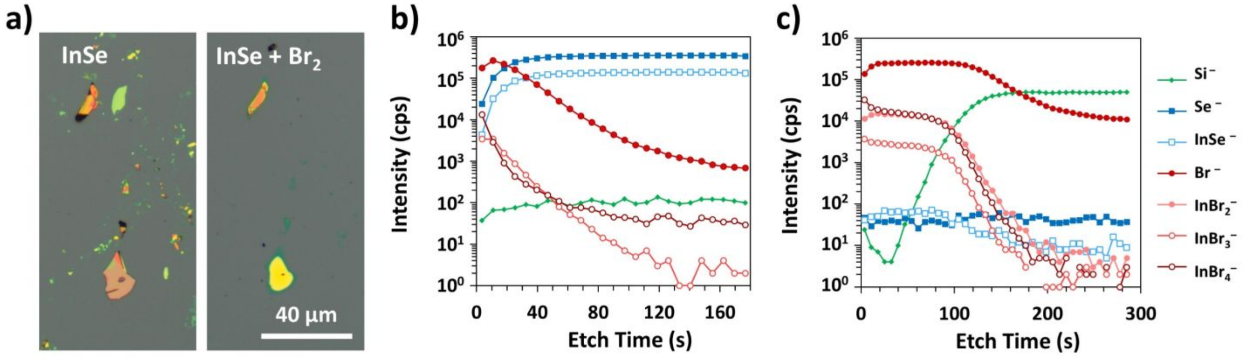

Figure S12. (a) Optical micrographs of mechanically exfoliated InSe flakes on Si/SiO_2 before and after treatment with Br_2. (b) ToF-SIMS depth profile of mechanically exfoliated InSe treated with I_2. (c) ToF-SIMS depth profile of c-InI_2 obtained by iodination of InSe (with PVP).

8. Morphotaxial Halogenation of In_2Se_3

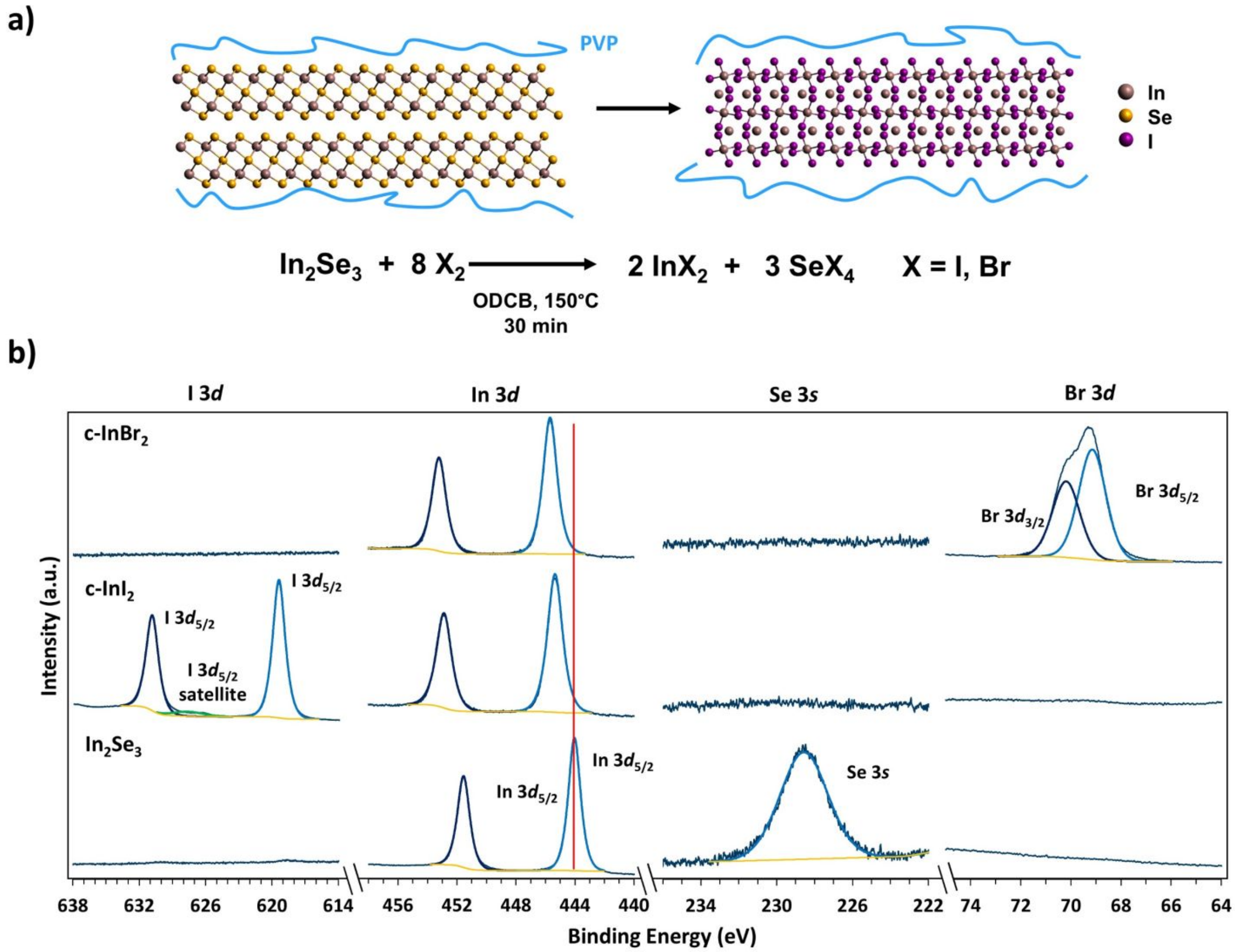

Figure S13. (a) Schematic of the morphotaxial halogenation of In_2Se_3. (b) XPS elemental peaks for electrochemically exfoliated In_2Se_3 and morphotaxial indium halides (c-InI_2 and c-$InBr_2$) obtained by halogenation of In_2Se_3. Binding energy scales are referenced to the substrate, which is an evaporated Au film on Si/SiO_2 (Au $4f_{7/2}$ = 84.0 eV). The Se 3*s* peak is shown because the Se 3*d* peak, which is more commonly used, overlaps with the Au $5p_{3/2}$ peak. The spectra are shifted vertically for clarity.

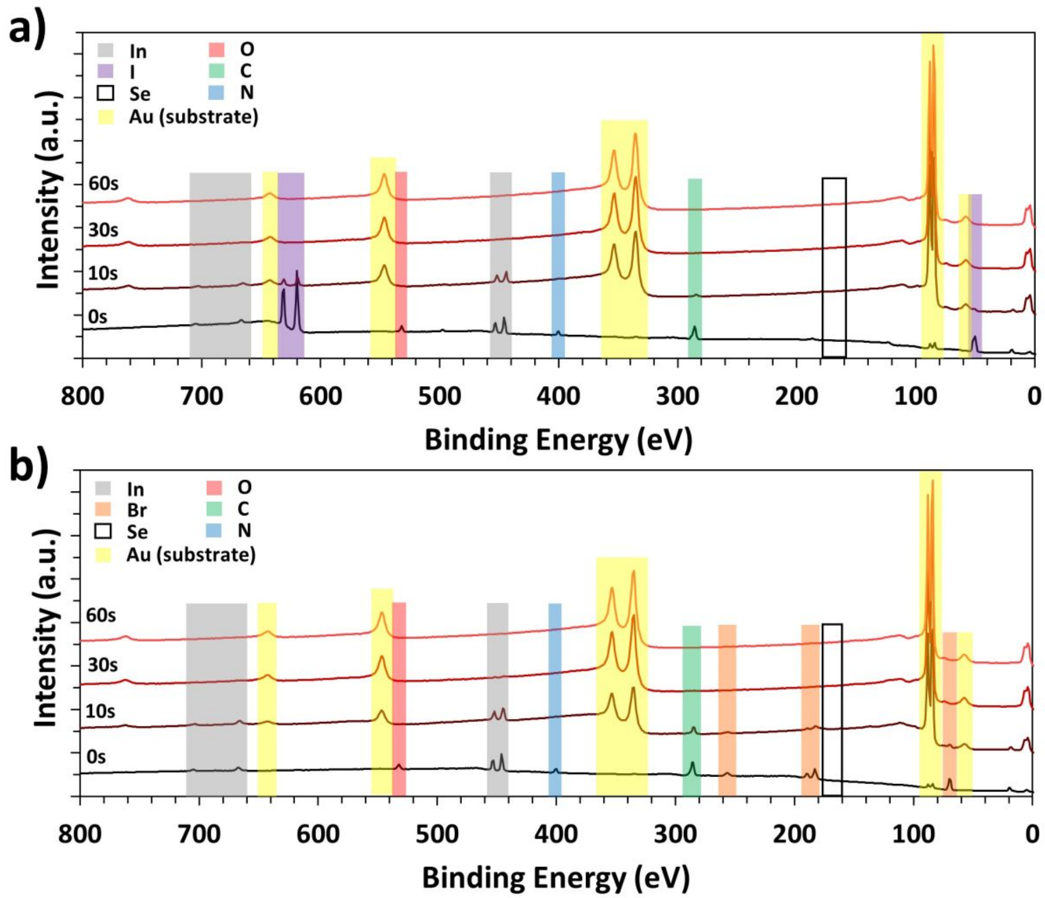

Figure S14. XPS depth profiles of (a) c-InI_2 and (b) c-$InBr_2$ obtained by halogenation of electrochemically exfoliated In_2Se_3. Binding energy scales are referenced to the substrate, which is an evaporated Au film on Si/SiO_2 (Au $4f_{7/2}$ = 84.0 eV). The spectra are shifted vertically for clarity.
